# Protocol to study human mitochondrial ribosome using quantitative density gradient analysis by mass spectrometry and complexome profiling analysis

**DOI:** 10.1016/j.xpro.2023.102605

**Published:** 2023-11-16

**Authors:** Petra Páleníková, Michal Minczuk, Pedro Rebelo-Guiomar

**Affiliations:** 1 MRC Mitochondrial Biology Unit, University of Cambridge, Hills Road, Cambridge CB2 0XY, UK

**Keywords:** Bioinformatics, Proteomics, Protein Expression and Purification

## Abstract

Dynamic macromolecular complexes containing a large number of components are often difficult to study using conventional approaches, such as immunoblotting. Here, we present a protocol for the analysis of macromolecular complexes in near-native conditions using a flexible setup to suit different cellular targets. We describe analysis of human mitochondrial ribosome, composed of 82 proteins, in a standardized way using density gradient ultracentrifugation coupled to quantitative mass spectrometry and subsequent analysis of the generated data (ComPrAn).

For complete details on the use and execution of this protocol, please refer to Páleníková et al.^1^ and Rebelo-Guiomar et al.^2^

## Highlights

• Standardized pipeline for analysis of mitochondrial ribosome assembly
• SILAC-based quantitative comparison of protein abundance
• Protocol can be adapted for the study of other macromolecular complexes
• Interactive tool for analysis of SILAC complexome profiling and qDGMS data

## Abstract (editor highlights)

Publisher’s note: Undertaking any experimental protocol requires adherence to local institutional guidelines for laboratory safety and ethics.

## Graphical abstract

**Figure:**
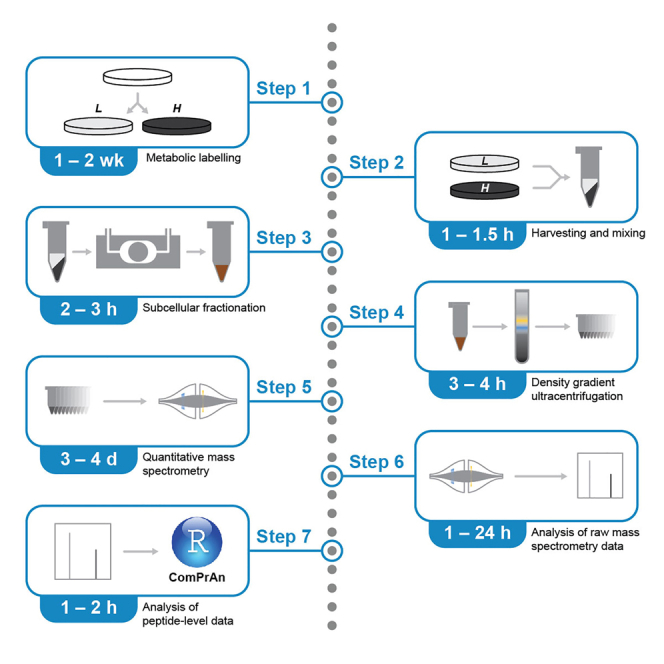

## Before you begin

The protocol below is adapted for the use of HEK 293 cells. However, we have also successfully used this protocol for HeLa, 143B and Hap1 cells. In this protocol, the step/discontinuous density gradient is performed in 14 mL SW 40 Ti tubes; we have also successfully used 5 mL SW 60 Ti ultracentrifuge tubes. Here, the continuous density gradient is performed in 2.2 mL TLS-55 ultracentrifuge tubes; we have also successfully used 5 mL SW 60 Ti and 14 mL SW 40 Ti ultracentrifuge tubes. The protocol describes an experiment performed in duplicate, with reciprocal metabolic labeling of control and test samples.

### Equipment

**Timing: 3–24 h**

1. Clean plastic tubes that will come into contact with samples to be analyzed by mass spectrometry.
  a. Wash a sufficient number of ultracentrifuge and 1.5 mL microcentrifuge tubes by immersing them in 10% methanol in ultrapure water.
  b. Drain as much solution as possible.
  c. Allow the tubes to completely dry at room temperature (18°C–25°C), or using an oven set to low temperature (check the thermal limits of the materials).

***Note:*** Plastic tubes that come into contact with the sample to be submitted to mass spectrometric analysis are washed to remove as much dust and plasticizers as possible, as these may introduce contaminants that will be detected during the analysis of samples. Avoid long storage times unless storage conditions do not promote accumulation of contaminants.

### Cell culture

**Timing: 1–7 days**

2. Culture the cells to be analyzed (here designated as control, c, and test, t) in their regular culture medium. If cells were thawed, allow a few passages for recovery. Make sure cells are adapted to the culture conditions before starting the experiment. These criteria are dependent on the cell line used.
3. Once cells are adapted, prepare SILAC media.
  a. Pre-warm two 500 mL bottle of DMEM for SILAC, two 50 mL vials of dialyzed fetal bovine serum (FBS) from a commercial source (dialyzed with 10,000 MW cutoff against 0.15 M NaCl until glucose < 5 mg/dL) by placing them in a 37°C water bath.
  b. Prepare fresh or thaw two 5 mL vials of penicillin-streptomycin, a 500 μL aliquot of light arginine, heavy arginine, light lysine, heavy lysine and two aliquots of proline. Allow frozen aliquots to reach room temperature (18°C–25°C).
    **CRITICAL:** This protocol is compatible with the use of most culture media, and culture conditions. The SILAC media should be as similar to the medium in which the used cells are routinely cultured, with the difference of being devoid of arginine and lysine in their composition, including the supplementations (e.g. serum).
    ***Note:*** SILAC media are supplemented with excess L-proline to avoid the production of proline from arginine, which would reduce the availability of labelled arginine to be incorporated into proteins and alter the labelling pattern.
  c. Place the warmed solutions and two filtering units in a laminar flow hood.
  d. Prepare the light medium (here designated as *L*) by adding 500 mL of DMEM for SILAC, 50 mL of dialyzed FBS, 5 mL of penicillin-streptomycin, 500 μL of L-proline, 500 μL of light L-arginine, and 500 μL of light L-lysine to the cup of a filtering unit.
  e. Prepare the heavy medium (here designated as *H*) by adding 500 mL of DMEM for SILAC, 50 mL of dialyzed FBS, 5 mL of penicillin-streptomycin, 500 μL of L-proline, 500 μL of heavy L-arginine, and 500 μL of heavy L-lysine to the cup of a filtering unit.
  f. Connect the filtering unit to the low-pressure line and allow the contents of the cup to be filter-sterilized into the bottle. Troubleshooting 1
  g. Disconnect the low-pressure line, carefully disassemble the filtering unit, and cap the bottle.
    ***Alternatives:*** Filter sterilization of prepared *L* and *H* SILAC media is performed to remove any potential contamination from amino acid aliquots. Alternatively, if the aliquots are already sterile, sterilization of the supplemented media becomes optional.
    ***Note:*** Filter sterilized SILAC medium can be stored at 4°C for 6 months.

### Preparation of analytical tools

To use ComPrAn, the analytical tool described in this protocol, R must be installed. This is a free software and the instructions for installation can be found at https://www.r-project.org/.

The ComPrAn R package is available from Bioconductor, and can be downloaded and installed by entering the following commands in R.

if (!requireNamespace("BiocManager", quietly = TRUE))

install.packages("BiocManager")

BiocManager::install("ComPrAn")

## Key resources table

**Table:** 

REAGENT or RESOURCE	SOURCE	IDENTIFIER
**Antibodies**		

Rabbit polyclonal anti-uL3m	Proteintech	16584-1-AP
Rabbit polyclonal anti-uS17m	Proteintech	18881-1-AP
Goat anti-mouse IgG (H + L), HRP Conjugate	Promega	W4021
Goat anti-rabbit IgG (H + L), HRP Conjugate	Promega	W4011

**Chemicals, peptides, and recombinant proteins**		

L-arginine (light L-arginine)	Sigma-Aldrich	A8094
^13^C_6_,^15^N_4_-L-arginine (heavy L-arginine)	Sigma-Aldrich	608033
cOmplete, EDTA-free proteinase inhibitor cocktail	Roche	11873580001
Dialyzed fetal bovine serum	Sigma-Aldrich	F0392
DMEM for SILAC	Thermo Fisher Scientific	A33822
L-lysine (light L-lysine)	Sigma-Aldrich	L8662
^13^C_6_,^15^N_2_-L-lysine (heavy L-lysine)	Cambridge Isotope Laboratories	CNLM-291-H-0.5
L-proline	Sigma-Aldrich	P5607
Penicillin-streptomycin (5,000 U/mL)	Thermo Fisher Scientific	15070063
RNasin ribonuclease inhibitor	Promega	N211A

**Critical commercial assays**		

DC Protein Assay Kit II	Bio-Rad	5000112

**Experimental models: Cell lines**		

Flp-In T-REx 293 cell line	Thermo Fisher Scientific	R78007

**Software and algorithms**		

R	(R Core Team, 2020)	https://www.r-project.org/
ComPrAn	Páleníková et al.^1^	http://www.bioconductor.org/packages/release/bioc/html/ComPrAn.html
Proteome Discoverer 1.4	Thermo Fisher Scientific	N/A

**Other**		

Vacuum filter system, 500 mL capacity, PES 0.22 μm membrane	Corning	431097
Minisart NML Syringe Filters, 0.2 μm, Sterile	Sartorius	16534-K
Cell homogenizer	Isobiotec	N/A
14 mL, Open-Top Thinwall Ultra-Clear Tube, 14 × 95 mm	Beckman Coulter	344060
2.2 mL, Open-Top Thinwall Ultra-Clear Tube, 11 × 34 mm	Beckman Coulter	347356

## Materials and equipment

• Appropriate equipment to handle and culture mammalian cells (e.g., 15 cm dishes, T175 flasks, 5-layer flasks).
• Refrigerated benchtop microcentrifuge capable of reaching 10,000 g (e.g., 5430 R, Eppendorf).
• Refrigerated benchtop centrifuge capable of reaching 13,000 g (e.g., 5804 R, Eppendorf).
• Ultracentrifuge capable of reaching 85,200 g (e.g., Optima L-100 XP, Beckman Coulter), and SW 40 Ti rotor.
• Benchtop ultracentrifuge capable of reaching 100,000 g (e.g., Optima MAX-XP, Beckman Coulter), and TLS-55 rotor.
• Gradient Master (Biocomp).
• In-line nanoscale liquid chromatography system, e.g., Proxeon EASY-nLC1000 system equipped with an Acclaim PepMap 2 micron C18 reverse-phase column (Thermo Scientific), 50 μm internal diameter, 150 mm length.
• Electrospray tandem mass spectrometer capable of analyzing high complexity peptide mixtures with high resolution, for example Q-Exactive Plus Orbitrap mass analyzer (Thermo Scientific).

**Table:** Light medium

Reagent	Final concentration	Amount
DMEM for SILAC	N/A	500 mL
Dialyzed FBS	9% (v/v)	50 mL
Penicillin-streptomycin	1x	5 mL
L-Proline	180 μg mL^-1^	500 μL of 200 mg mL^-1^ stock
L-Arginine	62 μg mL^-1^	500 μL of 69.3 mg mL^-1^ stock
L-Lysine	131 μg mL^-1^	500 μL of 145.8 mg mL^-1^ stock
**Total**	**N/A**	**556.5 mL**

Filter-sterilize (vacuum filter system) and store at 4°C. Avoid long storage times (> 6 months).

**Table:** Heavy medium

Reagent	Final concentration	Amount
DMEM for SILAC	N/A	500 mL
Dialyzed FBS	9% (v/v)	50 mL
Penicillin-streptomycin	1x	5 mL
L-Proline	180 μg mL^-1^	500 μL of 200 mg mL^-1^ stock
^13^C_6_,^15^N_4_-L-Arginine	79 μg mL^-1^	500 μL of 87.8 mg mL^-1^ stock
^13^C_6_,^15^N_2_-L-Lysine	137 μg mL^-1^	500 μL of 152.1 mg mL^-1^ stock
**Total**	**N/A**	**556.5 mL**

Filter-sterilize (vacuum filter system) and store at 4°C. Avoid long storage times (> 6 months).

**Table:** Hypotonic buffer

Reagent	Final concentration	Amount
HEPES, pH 7.8	20 mM	1 mL of 1 M stock
KCl	5 mM	250 μL of 1 M stock
MgCl_2_	1.5 mM	75 μL of 1 M stock
BSA	1 mg mL^-1^	50 mg
Protease inhibitor	1x	1 tablet
ddH_2_O	N/A	to 50 mL
**Total**	**N/A**	**50 mL**

Filter-sterilize (syringe filter unit) and store at 4°C.

**Table:** 2.5x MSH

Reagent	Final concentration	Amount
HEPES, pH 7.8	50 mM	5 mL of 1 M stock
Mannitol	525 mM	9.6 g
Sucrose	175 mM	6 g
EDTA	5 mM	1 mL of 500 mM stock
Protease inhibitor	2.5x	5 tablets
ddH_2_O	N/A	to 100 mL
**Total**	**N/A**	**100 mL**

Filter-sterilize (syringe filter unit) and store at 4°C.

**Table:** 1x MSH

Reagent	Final concentration	Amount
HEPES, pH 7.8	20 mM	2 mL of 1 M stock
Mannitol	210 mM	3.8 g
Sucrose	70 mM	2.4 g
EDTA	2 mM	400 μL of 500 mM stock
Protease inhibitor	1x	2 tablets
ddH_2_O	N/A	to 100 mL
**Total**	**N/A**	**100 mL**

Filter-sterilize (syringe filter unit) and store at 4°C.

**Table:** 1.5 M sucrose buffer

Reagent	Final concentration	Amount
HEPES, pH 7.8	10 mM	100 μL of 1 M stock
EDTA	5 mM	100 μL of 500 mM stock
Sucrose	1.5 M	5.1 g
ddH_2_O	N/A	to 10 mL
**Total**	**N/A**	**10 mL**

Filter-sterilize (syringe filter unit) and store at 4°C.

**Table:** 1.0 M sucrose buffer

Reagent	Final concentration	Amount
HEPES, pH 7.8	10 mM	100 μL of 1 M stock
EDTA	5 mM	100 μL of 500 mM stock
Sucrose	1.0 M	3.4 g
ddH_2_O	N/A	to 10 mL
**Total**	**N/A**	**10 mL**

Filter-sterilize (syringe filter unit) and store at 4°C.

**Table:** 0.5 M sucrose buffer

Reagent	Final concentration	Amount
HEPES, pH 7.8	10 mM	100 μL of 1 M stock
EDTA	5 mM	100 μL of 500 mM stock
Sucrose	0.5 M	1.7 g
ddH_2_O	N/A	to 10 mL
**Total**	**N/A**	**10 mL**

Filter-sterilize (syringe filter unit) and store at 4°C.

**Table:** Lysis buffer

Reagent	Final concentration	Amount
Tris-HCl, pH 7.4	50 mM	2.5 mL of 1 M stock
NaCl	150 mM	1.5 mL of 5 M stock
EDTA	1 mM	100 μL of 500 mM stock
Triton X-100	1% (v/v)	500 μL
Protease inhibitor	1x	1 tablet
RNase inhibitor	1 U μL^-1^	0.025 μL per 1 μL of buffer
ddH_2_O	N/A	to 50 mL
**Total**	**N/A**	**50 mL**

Filter-sterilize (syringe filter unit), aliquot, and store at 4°C. Add RNase inhibitor just prior to use.

**Table:** 10% (w/v) sucrose-TNM (Tris-NaCl-MgCl_2_)

Reagent	Final concentration	Amount
Tris-HCl, pH 7.4	50 mM	500 μL of 1 M stock
NaCl	100 mM	200 μL of 5 M stock
MgCl_2_	20 mM	200 μL of 1 M stock
Sucrose	10% (w/v)	1 g
ddH_2_O	N/A	to 10 mL
**Total**	**N/A**	**10 mL**

Filter-sterilize (syringe filter unit) and store at 4°C.

**Table:** 30% (w/v) sucrose-TNM

Reagent	Final concentration	Amount
Tris-HCl, pH 7.4	50 mM	500 μL of 1 M stock
NaCl	100 mM	200 μL of 5 M stock
MgCl_2_	20 mM	200 μL of 1 M stock
Sucrose	30% (w/v)	3 g
ddH_2_O	N/A	to 10 mL
**Total**	**N/A**	**10 mL**

Filter-sterilize (syringe filter unit) and store at 4°C.

## Step-by-step method details

### Metabolic labeling

**Timing: 1–2 weeks**

To allow quantitative comparison of cell proteomes between the two studied conditions, cell populations are expanded in the presence of amino acids containing different stable isotopes until most (≥95%) of their proteins have incorporated them.

1. Harvest cells of the two lines being analyzed and split them equally in two tubes.
2. Pellet cells by centrifugation at 500 *g* for 3 min.
3. Resuspend half of the control cell pellet with light SILAC medium, and the other half with heavy SILAC medium. Apply the same procedure for the test cell pellet. This will result in a total of 4 samples (i.e., c*L*, c*H*, t*L*, t*H*) that correspond to two replicates of the experiment with reciprocal labeling (i.e., c*L*t*H* and c*H*t*L*).

***Alternatives:*** It is highly recommended to perform this experiment with reciprocal labelling, i.e. have both control and test cell lines separately labelled with light and heavy isotopes, so that they are mixed in two different samples in which the only difference is the order of labelling. However, for preliminary experiments, and given the cost of amino acids containing heavy isotopes, it is possible to use a single labelling, with faster growing lines (typically control cells) being labelled with heavy medium and the other line with light medium. This way, the heavy medium is more effectively used to label cells, as incorporation goes to higher completion more quickly, and fewer medium exchanges are required.

4. Plate the cells in an adequate vessel, such as a 6-well plate or a T75 flask.

***Note:*** The number of cells used to start the labelling depends on their doubling time. For fast growing cells such as HEK 293 or HeLa (doubling time approx. 24 h), it may be convenient to start with low number of cells (e.g. 0.3 × 10^6^) as the population will expand during the labelling procedure according to $N=N_{0}e^{t\;\ln\left(2\right)/\tau }$, where $N_{0}$ is the cells seeded and τ the doubling time. If enrichment of a subcellular compartment is required, its proportion should be considered (e.g. if mitochondria are to be isolated and the cell line of interest has a low amount of these, then a larger number of cells are needed). Typically, 12-15 15 cm plates of HEK 293 cells for each labelling condition are sufficient for one experiment with mitochondrial isolation.

5. Allow the cell populations to expand in their respective SILAC medium for at least 7 doubling times, refreshing the medium every 2 days.

**CRITICAL:** It is crucial that the majority of the cell’s proteins are properly labelled. Since the regular culture medium contain naturally occurring isotopes, which are mostly the light counterparts, the labelling completion becomes an issue only for the populations incubated in the heavy medium. If the appropriate time to achieve >95% labelling has not been established, it is possible to monitor heavy isotope incorporation into cellular proteins by taking a sample while passaging and analyzing the whole cell lysate by mass spectrometry.

***Note:*** It is suggested that the amount of SILAC medium needed is determined before starting the experiment, to make sure there are enough resources available. Strategies to minimize the use of these could involve starting the labeling with a small number of cells and, attending to their doubling time, splitting them throughout labeling to obtain the desired number of cells at the end of this procedure.

### Harvesting and sample mixing

**Timing: 60–90 min**

**Figure 1:**
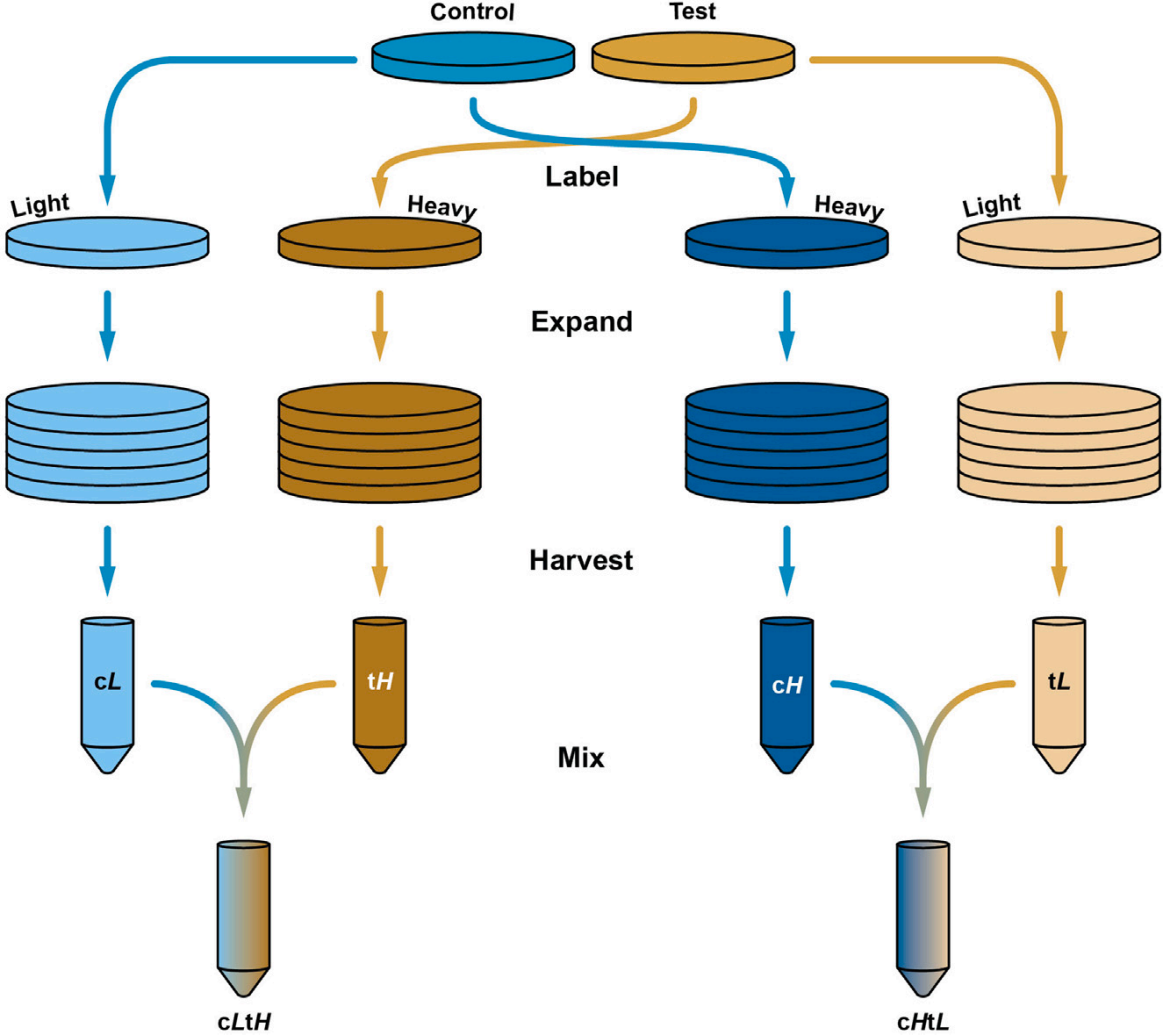
Schematics of sample mixing Control (c) and test (t) cells are separately labeled with SILAC media containing amino acids labeled with light (*L*) and heavy (*H*) isotopes. It is preferable that all cells are labeled simultaneously, but it is also possible to prepare one half of the full experimental set of cells at a time (i.e., c*L* and t*H*, or c*H* and t*L*). After expanding the cell populations, they are harvested and total protein quantified in each sample. The volume corresponding to the same amount of total protein in each pair is determined and mixed (i.e., c*L*t*H*, or c*H*t*L*).

Once sufficient (>95%) labeling of cells is achieved, labeled cells are collected and mixed. Mixing of cell suspensions in equal proportions is one of the most crucial steps in the protocol. Refer to Figure 1 for schematic of sample mixing.

6. The day before cell harvesting, place all required solutions, ultracentrifuge rotors and homogenizer at 4°C overnight.
7. On the day of the experiment, refrigerate the centrifuges and ultracentrifuges.
8. Harvest each cell population (c*L*, c*H*, t*L*, t*H*) into separate tubes.
  a. Adherent cell lines: detach the monolayer using trypsin or another routine dissociation method.
  b. Semi-adherent cell lines (e.g., HEK 293): detach the monolayer by pipetting directly on the monolayer.
  c. Suspension cell lines: centrifuge the suspension.
9. Retrieve cells from the suspension by centrifugation in a 50 mL conical tubes (500 *g*, 3–5 min, 4°C).
10. Discard the supernatant, resuspend the cell pellet in cold PBS, pellet cells, and discard supernatant.

***Note:*** Remove as much cell culture medium as possible as it contains proteins that may behave as contaminants in later stages of the procedure.

11. Resuspend the cell pellets in 50 mL conical tubes in 10 mL of cold PBS. Keep on ice.
12. Save a small fraction of cells labeled with SILAC heavy medium (i.e., c*H*, t*H*) to determine the extent of incorporation of heavy isotopes in cellular proteins.
  a. Resuspend the suspensions of cells labeled in heavy medium and immediately collect 10 μL into new, methanol washed, 1.5 mL microcentrifuge tubes.
  b. Pellet the sample in 1.5 mL tubes by centrifugation in a benchtop centrifuge (500 *g*, 3 min, 4°C) and carefully discard the supernatant.
  c. Freeze the cell pellets.

**CRITICAL:** It is important to store a small aliquot of heavy samples before sample mixing. These will be analyzed by mass spectrometry and inform on the extent of the isotopic labelling by the SILAC heavy medium. This information can be used to optimize the labelling time. If the labelling is determined to be insufficient, the quantitative comparison between samples cannot be made, as the unlabeled pool of proteins from heavy-labelled cells will be indistinguishable from that originating from light-labelled cells.

13. Determine protein concentration of heavy and light labeled samples.
  a. Resuspend cell suspensions of heavy and light labeled cells and immediately collect 10 μL of each into separate, new microcentrifuge tubes. Keep cell suspension in 50 mL conical tubes on ice.
  b. Pellet samples in microcentrifuge tubes by centrifugation in a benchtop centrifuge (500 *g*, 3 min, 4°C) and carefully discard the supernatant.
  c. Add 10–20 μL of lysis buffer and mix vigorously by pipetting up-and-down. Avoid foaming.
    **CRITICAL:** Although it is possible to use most protein quantification assays that provide good results for low concentrations, it is important to use a lysis buffer compatible with the assay. Check the interfering compounds (e.g. detergents, reducing agents) of the assay in advance to choose the adequate lysis buffer composition.
  d. Clarify the lysate by centrifugation in a benchtop centrifuge (15,000 *g*, 5 min, 4°C).
  e. Carefully collect the supernatant into a new tube, avoiding transferring pelleted debris or chromatin.
  f. Using a protein quantification of choice, determine the total protein concentration for each sample.
14. Identify the sample with the lowest total protein amount for the samples that will make up each mix (i.e., c*L*t*H* and c*H*t*L*), and determine the volume of the other counterpart that contains the same quantity of total protein.
15. Mix cell suspensions so that total protein quantities from each pair of control and test cell lines are the same.
16. Pellet cell mixes by centrifugation in a benchtop centrifuge (500 *g*, 3 min, 4°C) and carefully discard the supernatant. Keep the pellet on ice.

**CRITICAL:** It is important to keep cells on ice and perform all centrifugation steps at 4°C to limit degradation of biomolecules.

### Subcellular fractionation

**Timing: 2–3 h**

This section describes purification of cellular components in order to enrich samples for the macromolecular complex of interest while simultaneously reducing contaminating species. Macromolecular complex refers to a stable, functional multi-component unit of proteins that can also contain non-protein molecules, such as nucleic acids.

***Note:*** This step can be tailored to the target of the study. Here, we describe the purification of mitochondria by differential centrifugation and a step/discontinuous density gradient.

***Note:*** Solutions used in this and following sections can be stored at 4°C for up to 1 year if protease and RNase inhibitors have not been added. For solutions containing carbohydrates, storage conditions will depend on their sterility.

17. Weight the wet cell pellet in 50 mL conical tubes, using an empty tube as reference.
18. Gently resuspend the pellet in 3 mL of hypotonic buffer per 1 g of wet cell mass, and incubate on ice for 10 min. The hypotonic buffer causes cell swelling hence more efficient lysis upon homogenization (see next step).
19. Assemble the Balch homogenizer with a suitable ball bearing (12 μm clearance for HEK 293 cells) and wash the inner chamber with hypotonic buffer using two 2.5 mL syringes.

**CRITICAL:** Use a ball bearing with an appropriate clearance in the homogenizer – it must be just slightly smaller than the size of the used cells when in suspension. This may be verified using an optic microscope.

***Note:*** Make sure there are no air bubbles in the syringes or the inner chamber, as these will make the flow of the cell suspension more difficult.

20. Homogenize the cell suspensions.
  a. Pass 2 mL of cell suspension through the homogenizer, 3 times or until cells have been disrupted. Keep the homogenizer on ice at all times.
    ***Note:*** The weakest point of the Balch homogenizer is the connection with the syringes (Figure 2). Make sure to wear appropriate personal protective equipment, including goggles, and avoid applying excessive pressure on the plungers.
    ***Note:*** The number of passages required to homogenize cells may require optimization. This would involve looking at the homogenate under a light microscope and proceed with further passages if there are still a considerable number of undisrupted cells in the suspension.

**Figure 2:**
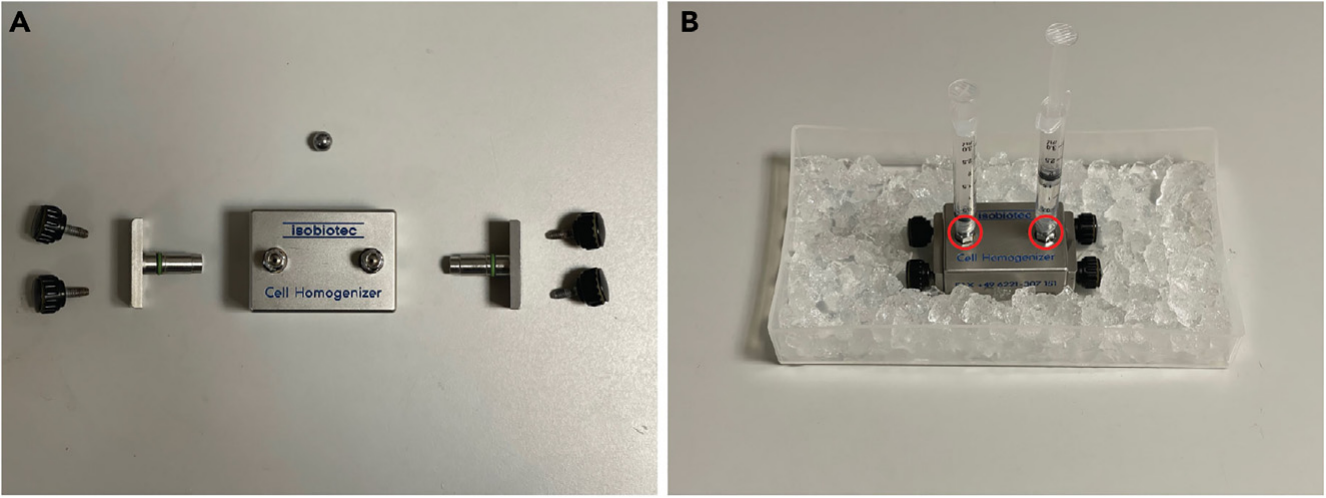
The assembly and use of Balch homogenizer (A and B) Parts of Balch homogenizer (A) and assembled homogenizer (B). The weakest point of the homogenizer is the connection with the syringes (red circles).
  b. Transfer the homogenate to a new 50 mL conical tube on ice, and immediately add 1.33 mL of 2.5x MSH.
    **CRITICAL:** Avoid keeping the homogenate in hypotonic buffer for too long. If possible, before starting homogenization, add the required volume of 2.5x MSH to the pre-chilled tube where the homogenate will be dispensed.
  c. Repeat the homogenization steps until all suspension has been used.
  d. Wash the homogenization chamber with 2.5x MSH and transfer the collected suspension to the tube containing the homogenate.
  e. Make the volume of the homogenate to 30 mL with 1x MSH.
  f. If processing more samples (e.g., both c*L*t*H* and c*H*t*L)*, disassemble the homogenizer, wash it in water and then 70% ethanol, and re-assemble it.
  g. Repeat homogenization for the next cell mix.
    ***Alternatives:*** The same procedure can be done using a Dounce homogenizer, or other preferred method for cell disruption. The main two points to consider are the efficiency of disruption and the integrity of the cell compartment of interest.
21. Remove debris from the homogenate by centrifugation (1,000 *g*, 20 min, 4°C).
22. Transfer the supernatant into a new 50 mL conical tube and keep on ice.
23. Pellet mitochondria by centrifugation (10,000 *g*, 20 min, 4°C). Troubleshooting 2
24. Discard the supernatant and resuspend the crude mitochondrial pellet in 1 mL of 1x MSH.
25. Prepare the step density gradient to purify mitochondria.
  a. Pipette 4 mL of 1.5 M sucrose buffer in a SW 40 Ti ultracentrifuge tube.
  b. Gently layer 4 mL of 1.0 M sucrose buffer on top.
  c. Gently layer 3 mL of 0.5 M sucrose buffer on top of previous layer.
  d. Layer the crude mitochondrial suspension on top of the gradient column.
  e. Top up the tube with 1 mL of 1x MSH.
26. Balance the tubes, and ultracentrifuge (85,200 *g*, 1 h, 4°C).
27. Locate mitochondria in the gradient – they should appear as a brownish-red disc between the 1.0 M and 1.5 M sucrose cushions (Figure 3).

**Figure 3:**
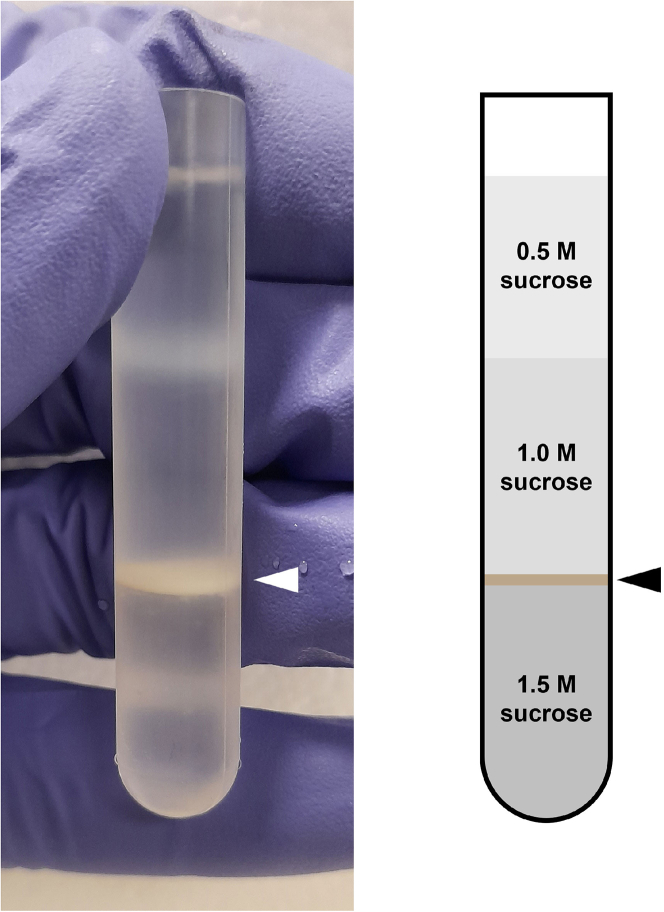
Expected results from purification of mitochondria by step/discontinuous density gradient ultracentrifugation The photograph on the left was taken after ultracentrifugation finished, and the purified mitochondrial disc is highlighted by a white arrowhead. On the right, a scheme of the tube contents, with the location of the purified mitochondria disc highlighted by a black arrowhead. The tube shown in this figure is a 5 mL SW 60 Ti ultracentrifuge tube.
28. Remove the 0.5 M and most of the 1.0 M layers by pipetting.
29. Collect the mitochondria by pipetting and transfer them to a 15 mL conical tube.
30. Resuspend the mitochondrial pellet in 4 volumes of 1x MSH.
31. Pellet the purified mitochondria by centrifugation (10,000 *g*, 10 min, 4°C). Discard the supernatant. Troubleshooting 2

### Continuous density gradient ultracentrifugation

**Timing: 3–4 h**

In this step, macromolecular complexes are separated by ultracentrifugation through a continuous density sucrose gradient. The conditions described here have been optimized for purification of mitochondrial ribosomes.

***Note:*** Separation is done according to the density of particles. Depending on the buffer used, it is possible to retrieve ribosomal subunits, or monosomes. This step is highly customizable and can be tailored to isolate other macromolecular complexes.

32. Lyse purified mitochondria.
  a. Add 250 μL of lysis buffer to the mitochondrial pellet.
  b. Place on a roller at 4°C for 15–20 min.
33. Meanwhile, prepare the continuous density gradient.
  a. Prepare the 10% (w/v) and 30% (w/v) sucrose-TNM (Tris-NaCl-MgCl_2_), 2.5 mL of each solution per pair of gradients, and keep on ice.
  b. Mark pairs of methanol-washed TLS-55 ultracentrifuge tubes using the appropriate block.
  c. Fill each tube to the mark with 10% (w/v) sucrose-TNM.
  d. Load 2.5 mL of 30% (w/v) sucrose-TNM into a syringe equipped with a blunt needle.
  e. Insert the needle to the bottom of the ultracentrifuge tube and load the solution from the bottom of the column, until the interphase reaches the mark.
  f. Cap the tubes and carefully place them on the magnetic base of the gradient maker.
  g. Run the TLS-55 10–30 Sucrose Short program on the gradient maker.
  h. Gently remove the tubes and keep them at 4°C until needed, limiting the manipulation of the tubes to avoid disruption of the formed continuous gradient.
34. Clarify the mitochondrial lysate by centrifugation (13,000 *g*, 5 min, 4°C).
35. Remove 100 μL from the top of the gradient column in the ultracentrifugation tube and load 200 μL of clarified mitochondrial lysate by dispensing slowly against the inner wall of the tube.
36. Balance the tubes, and ultracentrifuge (100,000 *g*, 2 h 15 min, 4°C).

***Note:*** The amount of material loaded on the continuous density gradient may require optimization. Avoid overloading the column (e.g. >2.5 mg of protein or >200 μL of sample in a TLS-55 ultracentrifuge tube) in order to obtain higher resolution of separation.

***Alternatives:*** The buffer composition, sucrose concentration and ultracentrifuge rotor can be adjusted to better suit the macromolecular complex being isolated.

37. Fractionate gradients (e.g., 100 μL per fraction) into methanol-washed 1.5 mL microcentrifuge tubes.

***Alternatives:*** Fractionation can be performed manually or automatically, from the top or the bottom of the tube. The smaller the volume of each fraction, the higher the resolution of the fractionation, but the probability of introducing technical artefacts is also higher.

***Optional:*** Transfer 5–10 μL of each fraction into new tubes for SDS-PAGE analysis. This will inform on the integrity of the gradient, and the distribution of the complex across fractions.

**Pause point:** Fractions can be stored at -80°C for several months until needed for mass spectrometric analysis.

### Quantitative mass spectrometry

**Timing: 3–4 days**

Proteins in liquid fractions from the density gradient are precipitated, digested, and processed for mass spectrometric analysis using a quantitative pipeline for SILAC. The duration of this step will depend on the number of fractions collected, and the pre-processing employed. The output of this step is a list of identified proteins, and their respective abundance between samples and across density gradient fractions.

38. Transfer an adequate volume of sample (e.g., 30 μL) to washed 2 mL microcentrifuge tubes.

***Note:*** The amount of material required for mass spectrometric analysis depends on the amount of total protein loaded on the density gradient, the amount of complex retrieved from it and the sensitivity of the mass spectrometric analysis used. In doubt, use as much volume as possible, as the processed material can be stored and used for additional mass spectrometric analyses.

39. Add 20 vol of cold ethanol to each fraction and incubate overnight (12–16 h) at -20°C.

***Note:*** Ethanol precipitation does not modify the protein residues while efficiently removing detergents with minimal protein loss, as well as allowing the easier resolubilisation of the precipitate. Alternative methods to concentrate proteins can be used.

40. Pellet the precipitate by centrifugation (16,000 *g*, 5 min, 4°C).
41. Add 1% (w/w) trypsin in 50 mM NH_4_HCO_3_ to the pellets and incubate overnight (12–16 h) at 37°C.
42. Fractionate the obtained peptide digests by nano-scale reverse-phase liquid chromatography using a gradient of 5–40% acetonitrile in 0.1% (v/v) formic acid over 84 min, at a flow rate of 300 nL min^-1^. The eluate is transferred directly to the electrospray interface of the mass spectrometer.
43. Acquire data from 400 to 1,600 *m/z* for precursor ions and set the ten most abundant multiply charged precursor ions in each spectrum to be fragmented by HCD with nitrogen. Precursor and fragment ion spectra are acquired with resolutions of 70,000 and 17,500, respectively.

**CRITICAL:** The settings and instruments used for mass spectrometric analysis can be flexibly adjusted. The key point is that each fraction is processed identically across the gradient, including their sequential analysis to minimize the effect of variations in instrument performance on sample analysis.

### Analysis of raw mass spectrometry data

**Timing: 1–24 h**

In this step peptides are identified and quantified from raw mass spectrometry data. The setup of the analysis should not take more than 1 h with the analysis running for approximately 8–12 h depending on the number of analyzed fractions. At the end, a table of all detected peptides matched to proteins is produced.

44. Using the Proteome Discoverer Deamon Utility submit the raw mass spectrometry files for all fractions for analysis by Proteome Discoverer.
45. Identify peptides by comparing the MS/MS spectra to the appropriate Mascot database with Proteome Discoverer.
46. Quantify peptides using Proteome Discoverer.
47. Open a report from all fractions.
48. Export the report of all peptides from all fractions as a single tab delimited text file (please refer to ComPrAn help files). Make sure that “Peptides” box is checked under Criteria. The stem “psms” will be automatically added into the filename.
49. Use file from step 48 as an input for analysis by the ComPrAn R package.

***Note:*** For the analysis of samples from HEK 293 cells, we used following parameters: proteins were searched against human UniProt database; mass tolerances of 10 ppm and 0.5 Da were used for precursor ions and fragmented ions, respectively; trypsin was selected as the protease with one missed cleavage allowed; dynamic modifications included N-terminal acetylation and formylation, oxidation of methionine and propionamide modification of cysteine; Mascot was configured to consider the possibility of the presence of the heavy-labelled lysine and arginine.

### Analysis of peptide level data

**Timing: 1–2 h**

This section describes analysis of peptide level data with the use of ComPrAn R package. As an output, a table of normalized protein data, lists of proteins detected only in one sample, a table of clustered proteins, as well as various visualizations of results are created. Analysis steps are illustrated in Methods Video S1.

***Note:*** Two types of input data can be used in ComPrAn: i) peptide data or ii) normalized protein data. In this protocol steps 51 to 58 describe a workflow for processing peptide level data, that results in the generation of normalized protein data which are used from step 59 onwards.

***Note:*** ComPrAn contains internal example datasets: i) peptide dataset obtained with Proteome Discoverer version 1.4 (Thermo Fisher Scientific) in combination with Mascot database for peptide quantification and identification, respectively and ii) normalized protein dataset obtained by processing the example peptide dataset (i) in ComPrAn. These datasets can be accessed, and the first 6 rows viewed by entering following code in R:

# load ComPrAn package

library(ComPrAn)

# locate peptide dataset

inputFile <- system.file("extData", "data.txt", package = "ComPrAn")

# read in example peptide dataset using the designated

# ComPrAn import function

peptides <- peptideImport(inputFile)

# locate normalised protein dataset

inputFileProtein <- system.file("extData", "dataNormProts.txt",

package = "ComPrAn")

# read in file using the dedicated ComPrAn function, this

# function automatically changes the structure of the table to

# the required format

proteins <- protImportForAnalysis(inputFileProtein)

# display first 6 rows of peptides data

head(peptides)

# display first 6 rows of protein data

head(proteins)

50. Start R, load the ComPrAn package and launch the Shiny app by entering the following code.

# load ComPrAn R package

library(ComPrAn)

# launch the Shiny app

compranApp()

51. Import the data by clicking on ‘Import’ tab and ‘Browse…’ button; navigate to the file produced in step 48 and click Open.
52. After progress bar shows “Upload complete” click ‘Process data’ button.
53. Specify names of the labeled (“heavy”) and unlabeled (“light”) samples in the appropriate boxes (these names will be used in plots later).
54. Switch to the ‘Peptide-to-protein’ tab and click on ‘Summary’ section in the list that will appear.

***Note:*** Summary plots contain the information about total number of peptides and number of peptides in the two studied samples that passed the initial filtering (removes peptides which were not assigned into a protein group, had missing precursor area value or had a “Rejected” value of PSM (peptide spectrum match) ambiguity).

55. Switch to the ‘Filter and Select’ tab. Adjust the available setting and click ‘Filter the data’ button. Plots summarizing total number of proteins in samples are shown.
56. In the same tab, click ‘Select peptides’ button to select representative peptides that will represent each protein in the following analysis.
57. Switch to ‘Rep Peptides’ tab.

***Note:*** On this page all peptides for any given protein can be visualized with the option to highlight the peptide that was selected by the analysis software as representative of the protein. Additionally, lists of proteins that were detected in both or only one of the two samples can be downloaded.

58. Switch to ‘Normalize’ tab. Click on ‘Normalize the data’ button; all protein quantity values will be normalized to be between 0 and 1, a progress bar will appear in the bottom right corner, indicating processing of the data, once finished a message “Part 1 analysis finished, you may proceed to Part 2” will be shown.

**Pause point:** normalized data can be exported in tab delimited format and used as alternative input to continue with analysis straight from step 59.

59. Switch to ‘Protein workflow’ and click on ‘Normalized Proteins’ tab in the list that will appear. A protein can be selected from drop down menu for visualization of its quantitative comparison between labeled and unlabeled samples (Figures 5).

***Note:*** Each section of ‘Protein workflow’ provides a visualization option for the protein-level data to compare proteins and protein complexes quantitatively or qualitatively. Multiple options for customization of plots are provided, and each plot can be exported as a pdf file.

60. Switch to ‘Heatmap’ tab, click on ‘Browse…’ and navigate to the file with information about the protein complex of interest. This produces a heatmap showing the quantitative comparison of protein profiles between samples (Figures 5).
61. Switch to ‘Co-migration plots’ tab, paste UniProt IDs of interest in a box to qualitatively compare the profile of one or two protein complexes.
62. Switch to ‘Cluster’ tab. This section provides functionality for clustering analysis. Proteins are assigned into clusters, separately for each sample, based on correlation of protein migration profiles. Plots showing the number of proteins per cluster and table with proteins assigned into clusters can be downloaded.

***Alternatives:*** The analysis described here with the use of ComPrAn app can also be performed directly from the R command line with the use of ComPrAn functions. For step-by-step guidelines use ComPrAn R package vignettes that can be accessed by entering following commands into R: This will open a web page with a list of available vignettes. Command line analysis of peptide-level data is described in a “SILAC complexomics” vignette and analysis of protein-level data is described in “Protein workflow” vignette.

browseVignettes("ComPrAn")

Methods Video S1. Walkthrough of ComPrAn analysis This video shows the analysis of peptide-level data and visualization of protein data as described in steps 50 to 62 of the protocol.

## Expected outcomes

**Figure 4:**
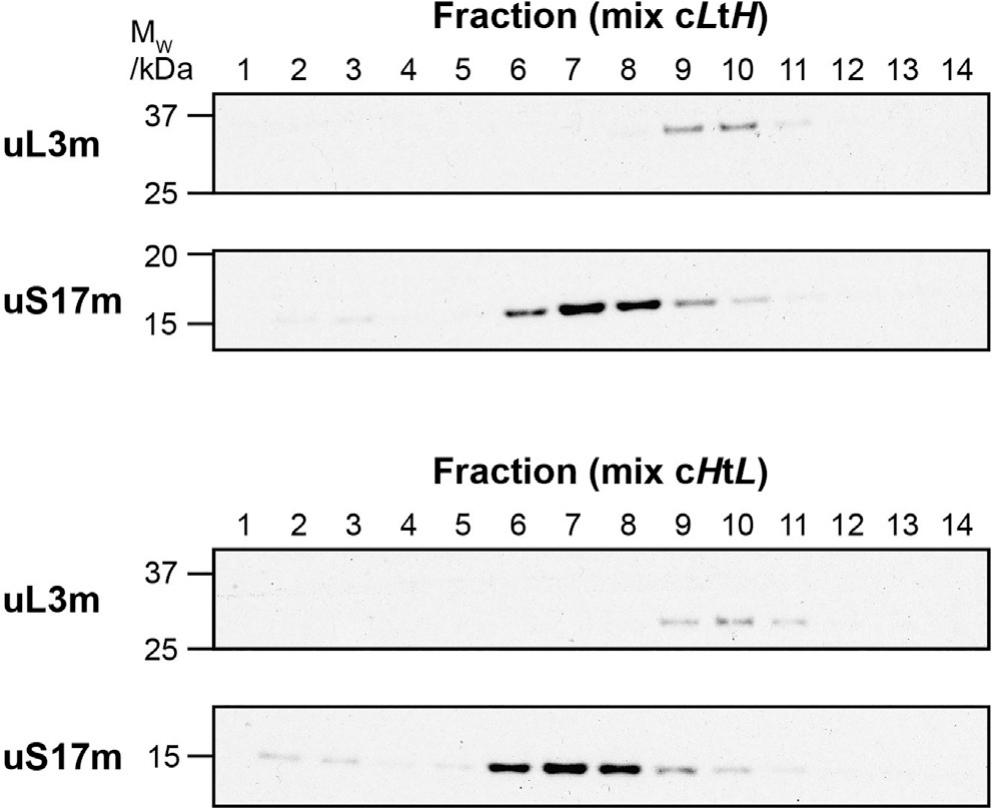
Expected results from isolation of mitoribosomal subunits by continuous density gradient ultracentrifugation Fractions were collected from the top of each gradient, their components separated by SDS-PAGE, and subsequently analyzed by immunoblotting against proteins of the large (uL3m) and small (uS17m) mitoribosomal subunits (primary antibodies used at 1:2000, secondary antibodies at 1:3000). In the presented case, control (c) and test (t) correspond to parental and MRM2 knock-out cells.^2^

**Figure 5:**
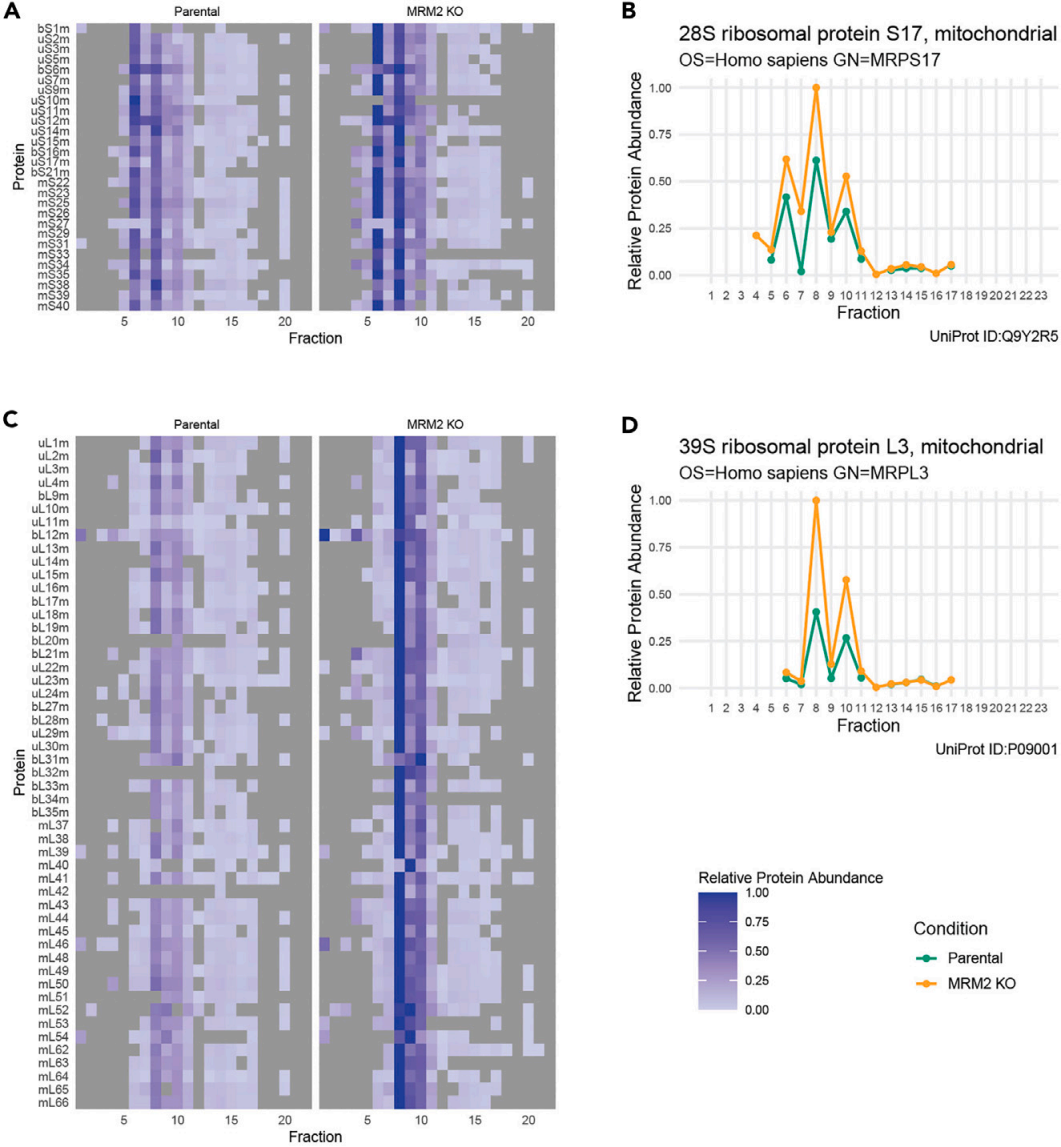
Example of plots obtained from ComPrAn Data from qDGMS comparing parental and MRM2 knock-out mitochondrial proteomes,^2^ highlighting the small (A, B) and large (C, D) mitoribosomal subunits. (A–D) Heatmaps can be produced in ComPrAn (A, C) for simultaneous visualization of relative protein abundance of multiple proteins, as well as single line/dot plots (B, D) to highlight the comparison of single proteins across both analyzed samples.

This protocol describes a complete experimental and computational pipeline for analysis of mitochondrial ribosome from mammalian cells. The amount of purified mitochondria varies greatly according to the cell line used, but also the conditions under which cells are (e.g., drug treatment, genetic engineering). HEK 293 cells yield approximately 10% of their weight in purified mitochondrial mass. Purified mitochondria are expected to be located between the 1.0 M and 1.5 M sucrose layers in the step/discontinuous gradient. These should appear as a thick brownish disc (Figure 3). Mitoribosomal subunits are expected to migrate 30–50% into the gradient. For 21 fractions of 100 μL each, the small subunit (mtSSU) is expected to be enriched in fractions 6-8, and the large subunit (mtLSU) in fractions 9-11 (Figure 4). Further examples of expected results can be obtained from Van Haute et al.^3^ (Figure 6 and Supplementary Dataset) and Rebelo-Guiomar et al.^2^ (Figure 4, Supplementary Figure 4), where the qDGMS methodology was employed. The output from ComPrAn includes normalized and clustered protein data as well as visualization of proteins and protein complexes in a form of line plots and heatmaps (Figure 5). Examples of ComPrAn output can be obtained from D’Souza et al.^4^ (Figure 6, Supplementary Figure 7, Supplementary Datasets 2 and 3).

## Limitations

Study of macromolecular complexes beyond mitochondrial ribosome with the methodology described in this protocol might require optimization. Nevertheless, we were able to analyze another mitochondrial complexes and two abundant cytosolic complexes with the experimental conditions presented here (Figure 8 in Páleníková et al.^1^). To achieve best results for other macromolecular complexes, including protein aggregates or membrane proteins, ideal conditions for complex separation need to be optimized for each macromolecular complex. This includes both composition of the continuous sucrose gradient as well as ultracentrifugation conditions. The method of gradient fractionation affects the resolution of the gradient, with automated systems providing better reproducibility and easier fractionation into higher number of fractions with smaller volume. Integral part of the protocol is SILAC labeling of the studied samples, which limits the use of the protocol to cell lines that can be grown in culture in sufficient quantities. SILAC labeling enables mixing of studied samples at early stage of the protocol, mitigating some technical variability. However, this limits the number of samples that can be analyzed in a single experiment, with 2 sample comparison described in this protocol.

The analytical pipeline described here does not process raw mass spectrometry data. Therefore, raw mass spectra need to be pre-processed in software that allows peptide identification and quantification. ComPrAn has been optimized to work with the output from Proteome Discoverer, and in case alternative software is used for processing of raw data a manual rearrangement of data might be necessary to acquire data in a format compatible with ComPrAn. Similar to the experimental pipeline, ComPrAn allows for direct analysis and visualization of only 2 biological samples.

## Troubleshooting

### Problem 1

When preparing SILAC media, the solutions are filtered very slowly.

### Potential solution

Confirm that the low pressure line is creating a sufficiently low pressure to allow the contents of the cup to be filtered. We found that typical liquid aspiration lines used routinely in cell culture provide low enough pressure for this task. This problem can also be related to the dialyzed FBS used, which, due to dialysis, may contain protein aggregates. These are often visible under an optic microscope, which can be initially used to test this hypothesis. If this is the case, centrifuge the dialyzed FBS before adding it to the medium to be filtered in order to remove the aggregates.

### Problem 2

The amount of purified cell compartment is low.

### Potential solution

Consider the relative proportion of the isolated compartment relative to the whole cell, and in the context of the cell type used. The homogenization step may have been inefficient, leaving a large proportion of cells intact, which were discarded in the first centrifugation step at low speed. If that is the case, retrieve the debris pellet and re-homogenize it. A potential cause for low yield may be the opposite – clearance was too low, homogenization was too vigorous or the conditions were not appropriate to preserve the compartment intact. Consider the use of protease and/or nuclease inhibitors during cell disruption. Finally, determine how pure the compartment of interest needs to be – additional processing steps may reduce yield.

## Resource availability

### Lead contact

Further information and requests for resources and reagents should be directed to and will be fulfilled by the lead contact, Michal Minczuk (michal.minczuk@mrc-mbu.cam.ac.uk).

### Materials availability

There were no new materials generated in this work.

## Data Availability

Complexome Profiling Analysis (ComPrAn) is freely available and can be installed from http://www.bioconductor.org/packages/release/bioc/html/ComPrAn.html.
